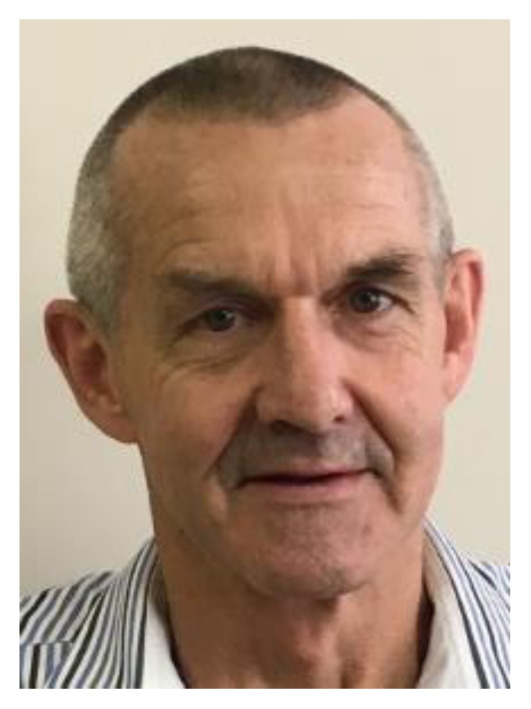# The state of the South African Journal of Sports Medicine, 2019

**DOI:** 10.17159/2078-516X/2019/v31i1a6055

**Published:** 2019-01-01

**Authors:** Mike Lambert

**Affiliations:** Editor-in-chief

This is the second year of the new format of the South African Journal of Sports Medicine. When we made the change in 2017 we were confident it was going to have a positive impact on the quality of the journal ^[1]^. The reason for making the change to a one-issue-per-year format was primarily to speed up the time from accepting a paper to the time of publication. We reported last year that this time was reduced to about three weeks ^[2]^. We received positive feedback from authors and can now report this duration has been reduced to two weeks in most cases.

We use the Open Journal System (OJS) as the journal management system for the South African Journal of Sports Medicine. OJS is open source software designed for the management of peer reviewed academic journals. This was developed by the Public Knowledge Project, a non-profit research initiative, through collaboration between the Faculty of Education at the University of British Columbia, the Canadian Centre for Studies in Publishing at Simon Fraser University, the University of Pittsburgh, Ontario Council of University Libraries, the California Digital Library and the School of Education at Stanford University ^[3]^. In 2018 the OJS was upgraded to version 3. This change improved the usability of the website for authors and reviewers. This version of the OJS also has an automated feature for exporting a citation to a reference manager. Altmetric data are also readily available for each publication.

The South African Journal of Sports Medicine remains accredited by the Scientific Electronic Library Online (SciELO) SA, South Africa’s main open-access (free to access and free to publish) searchable full-text journal database. SciELO SA is managed by the Academy of Science of South Africa (ASSAf), funded by the South African Department of Science and Technology and endorsed by the South African Department of Higher Education and Training (DHET).The SciELO SA database represents 76 journals published within South Africa. The South African Journal of Sports Medicine is also on the Department of Higher Education and Training (DHET) list of accredited journals. This is important because South African authors who work at tertiary institutions and publish in the South African Journal of Sports Medicine can get a subsidy for their paper from the DHET.

A requirement for 2019 is that all authors and co-authors have to include their Open Researcher and Contributor IDs **(**ORCid) when the manuscript is submitted. This seems to be an international trend in academic publishing and is designed to provide each author with unique identification. The ORCid can easily be obtained by registering on the ORCid website (http://www.orcid.org).

Our publishing team includes a journal manager, copyeditor and typesetter. This committed group has developed standard operating procedures which enable an efficient workflow from the submitted paper through to the published paper. It is noteworthy that this group operates within the constraints of an extremely limited annual budget.

However, we do have challenges. The major challenge, which we share with all journals, is to get quality reviews of submitted manuscripts. The peer review process, a fundamental principle of science, relies on experts who can share their time to review a paper. Because this is usually done anonymously, the reviewers do not get recognition. While it is always noble to make a contribution without recognition, the reality is that more experts are declining requests to review a paper. This increases the burden on editorial staff who have to spend more time trying to find a competent and willing reviewer. Publons (http://www.publons.com/about/home/) have attempted to address this problem by providing an online platform where reviews are recorded. Once a review is completed, the editor of the journal emails the reviewer acknowledging the submitted report. The reviewer simply forwards this email to the Publons platform where the information is recorded in the reviewer’s file. The platform can generate data rich reports on the numbers of reviews, quality of journals and ranking of the researcher. This profile provides the reviewer with useful information which can be used in a job promotion or funding application.

Finally, a reminder that the South African Journal of Sports Medicine is sponsored by the South African Sports Medicine Association (SASMA). Therefore we are obligated fulfil the objectives of SASMA and ensure that the publications are relevant to issues in South Africa.[Fig f1-2078-516x-31-v31i1a6055]

**Figure f1-2078-516x-31-v31i1a6055:**